# TMEM205 Is an Independent Prognostic Factor and Is Associated With Immune Cell Infiltrates in Hepatocellular Carcinoma

**DOI:** 10.3389/fgene.2020.575776

**Published:** 2020-10-14

**Authors:** Jiawei Rao, Xukun Wu, Xiaozhuan Zhou, Ronghai Deng, Yi Ma

**Affiliations:** ^1^Department of Organ Transplantation, First Affiliated Hospital of Sun Yat-sen University, Guangzhou, China; ^2^Department of Hepatology Surgery, First Affiliated Hospital of Sun Yat-sen University, Guangzhou, China; ^3^Department of Gastroenterology, First Affiliated Hospital of Sun Yat-sen University, Guangzhou, China

**Keywords:** tumor immunity, hepatocellular carcinoma (HCC), TMEM205, prognostic factor, the Cancer Genome Atlas (TCGA)

## Abstract

Hepatocellular carcinoma (HCC) is the second leading cause of cancer-related death worldwide despite the availability of diverse treatment strategies. Much research progress has been made regarding immunotherapy but the effects remain unsatisfactory, highlighting the urgent need for novel immune-related therapy targets. In recent years, more and more studies have pointed out the associations between certain transmembrane (TMEM) family proteins and tumor progression, but the role of TMEM205 remains unclear. In this study, we analyzed the RNA-seq and clinical data of 371 patients from The Cancer Genome Atlas (TCGA) and found significant differential expression of *TMEM205* between normal and tumor tissues (*P* < 0.001). Low *TMEM205* expression was also found to be independently associated with poor overall survival (OS; *p* = 0.032) and poor disease-specific survival (DSS; *p* = 0.002) in multivariate Cox regression analyses. RNA-seq and clinical data from hepatocellular carcinoma patients in the International Cancer Genome Consortium (ICGC) also showed significant differential expression of *TMEM205* (*P* < 0.001) and association between low *TMEM205* expression and poor survival (*P* < 0.001). We also used the Estimate the Proportion of Immune and Cancer cells (EPIC) tool to estimate the proportions of various immune cells in the tumor tissues. A correlation analysis was conducted, and *TMEM205* expression in tumor tissues was found to be significantly associated with the proportion of macrophages (Pearson *r* = 0.45, *p* < 0.0001). A negative correlation was found between *TMEM205* expression and M2 macrophage markers (*CD163*, *EGR2*, and *MS4A4A*) and between *TMEM205* expression and regulatory T cell (Treg) markers (*CCR8*, *STAT5B*, and *IL2RA*), while a positive correlation was found between *TMEM205* expression and the proportion of CD8+ T cells (Pearson *r* = 0.26, *p* < 0.0001). In conclusion, TMEM205 might improve HCC patients’ prognosis by reducing the levels of immunosuppressive cells (M2 macrophages and Tregs) and facilitating the infiltration of cytotoxic T cells into the tumor microenvironment. Therefore, TMEM205 has potential as a prognostic biomarker and immunotherapy agent in combination therapy regimens for HCC.

## Introduction

Hepatocellular carcinoma (HCC) is the second leading cause of cancer-related deaths worldwide, causing nearly 800,000 deaths every year (Ferlay et al., 2015). Most cases develop from cirrhosis, which is mainly associated with chronic infection with hepatitis B virus and hepatitis C virus.

Nowadays, diverse strategies are used to treat HCC, including surgery, liver transplantation, ablation, transhepatic arterial chemotherapy and embolization, and molecularly targeted therapy. Unfortunately, the prognosis of advanced HCC patients remains poor (Bruix et al., 2017; Finn et al., 2018). In the past few decades, increasing knowledge of immune-related mechanisms has led to the development of immunotherapy for HCC. Programmed death-1 (PD-1) is expressed on CD8+ and CD4+ T cells and, upon binding to its ligand PD-L1, which is expressed on many cancer cells, it inhibits T cell activation; it has therefore been considered an ideal target for immunotherapy. Although nivolumab (anti-PD-1) has shown some therapeutic effect as a second line treatment in advanced HCC (El-Khoueiry et al., 2017), its variable clinical efficacy and limited applications (in terms of the suitable patients) make its future use in HCC unclear (Zhu et al., 2018). Therefore, a novel immune-related therapy target in HCC is urgently needed.

In the tumor microenvironment (TME), the fate of tumor cells is often decided by the interactions with immune cells and stromal cells. Tumor-associated macrophages (TAMs) surprisingly occupy up to 50% of the tumor tissue and dominate the immune cells that infiltrate the TME (Tu et al., 2014; Van Overmeire et al., 2014). The M2-like phagocytic TAMs are induced by interleukin (IL)-4, IL-6, and IL-10 (Mantovani et al., 2004; Wang et al., 2010) and Notch. They can recruit regulatory T cells (Treg) to cancer sites via the expression of CCL17, CCL18, and CCL22, thereby blocking the activation of cytotoxic T cells (Mamrot et al., 2019; Wang et al., 2019). A positive feedback loop may exist between M2-like macrophages and Tregs, which can lead to severe immunosuppressive effects, affecting cancer progression. Additionally, mounting evidence has indicated that the presence of functionally exhausted T cells in tumor tissues may be associated with resistance to immunotherapy (Anderson et al., 2017; Chen and Mellman, 2017). The limited clinical effect of immunotherapy (e.g., anti-PD-1) may be related to disturbances in the immunosuppressive cells (such as increased M2-like macrophage levels) in the TME, and there is an urgent need for a detailed understanding of the TAM-mediated inhibition of cytotoxic T cells (Noy and Pollard, 2014).

The transmembrane (TMEM) family of proteins are proteins that span biological membranes, most of which extend through the lipid bilayer of the plasma membrane, while others are located in the membranes of organelles. Although the functions of most TMEM proteins remain unknown, increasing attention has been paid to the associations between TMEM and tumor initiation and progression. TMEM176A expression inhibited the tumor cell growth of esophageal squamous cell carcinoma both *in vitro* and *in vivo* (Wang et al., 2017), and a very similar study revealed that the TMEM176 promoter was methylated in nearly 50% of primary colorectal cancers (Gao et al., 2017). Additionally, TMEM48 overexpression in lung cancer was associated with lung cancer cell proliferation, migration, and invasion (Qiao et al., 2016). Furthermore, TMEM48 suppression led to increased expression of apoptotic and tumor suppressor proteins such as caspase 3, PTEN, and p53 (Akkafa et al., 2018).

TMEM205 has been found to be highly expressed in the liver (Shen et al., 2010), but its role in HCC has not previously been explored. Additionally, TMEM205 was found to be in the top five differentially expressed genes at 1 week and 1 month after kidney transplantation, which suggests that it may have a role in the immune response (Bueno et al., 2015). Using RNA-seq data and clinical data from The Cancer Genome Atlas (TCGA) and International Cancer Genome Consortium (ICGC), this study explored whether TMEM205 is a prognostic marker in HCC patients and the associations between TMEM205 expression and the proportions of different types of immune cells in the TME. We hope to provide new understanding of the immune microenvironment and insights into a potential immunotherapy target for future treatment of HCC.

## Materials and Methods

**FIGURE 1:**
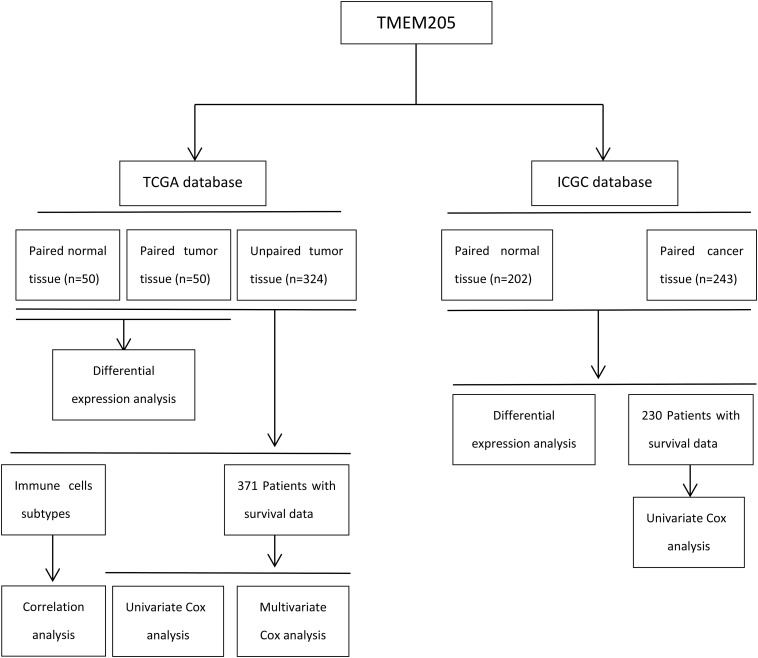
Flow chart of the study design.

Publicly available RNA-seq data (samples = 424) and complete clinical data from HCC patients (*n* = 371) were downloaded from TCGA. Six patients were excluded in TCGA because their survival time was 0. We also collected available RNA-seq data (samples = 445) and clinical data from HCC patients (*n* = 230) in ICGC. The proportions of various types of immune and stromal cells in the tumor tissues were estimated using RNA-seq data and the Estimate the Proportion of Immune and Cancer cells (EPIC) tool, which integrates novel marker expression profiles from non-malignant cell types found in tumors and involves renormalization based on the cell-type-specific mRNA levels^1^. Details of the steps are summarized in Figure 1 through a flowchart.

The statistical analysis was performed in R 3.6.3 (R Foundation for Statistical Computing, Vienna, Austria). The differential expression of *TMEM205* between normal and tumor tissues were visualized through ggboxplot function of “ggpubr” package. Kaplan–Meier analyses and Cox regression analyses (both univariate and multivariate) were performed using the “survival” and “survminer” packages in R, with *p* < 0.05 being considered to represent statistical significance (though not in the subsequent Pearson correlation analyses). The optimum cutpoint regarding low *TMEM205* expression was determined using the “surv_cutpoint” function in the “survminer” package in R. Pearson correlation analyses were conducted using the basic “cor.test” function, with only *p* < 0.0001 being considered to represent statistical significance.

Most of the images in this paper were generated using R. We used the “ggsurvplot” and “ggforest” functions in the “survminer” package to generate Kaplan–Meier survival curves and forest plots, respectively. The “ggscater” function in the “ggpubr” package and the “corrplot” function in the “corrplot” package were used to visualize the correlation analyses. The estimated proportions of various types of immune and stromal cells in the tumor tissues were plotted in pie chart using Microsoft Excel 2010.

## Results

### Differentially Expressed *TMEM205* Is an Independent Prognostic Factor in HCC Patients

**FIGURE 2:**
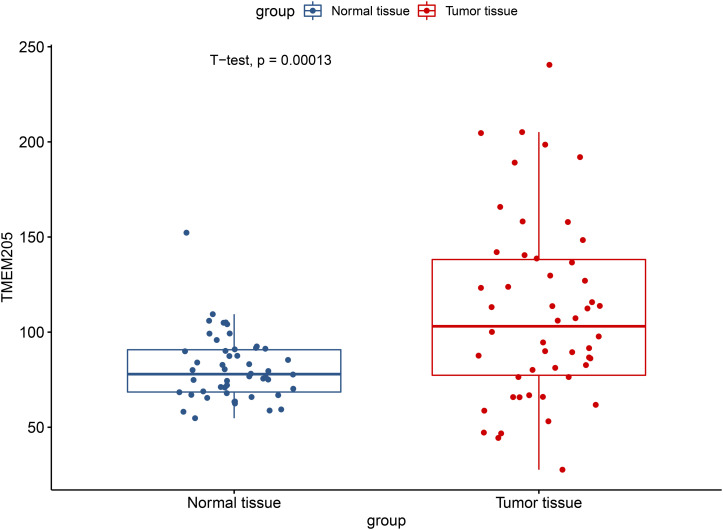
Differential expression of TMEM205 between normal and tumor tissues in TCGA database.

**FIGURE 3:**
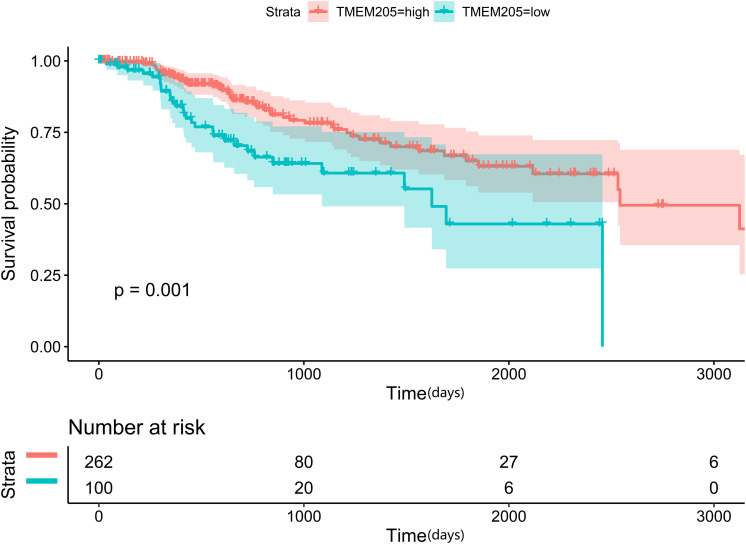
Low expression of TMEM205 is predictive for poor disease-specific survival (DSS) in hepatocellular carcinoma (HCC) patients from TCGA database.

**FIGURE 4:**
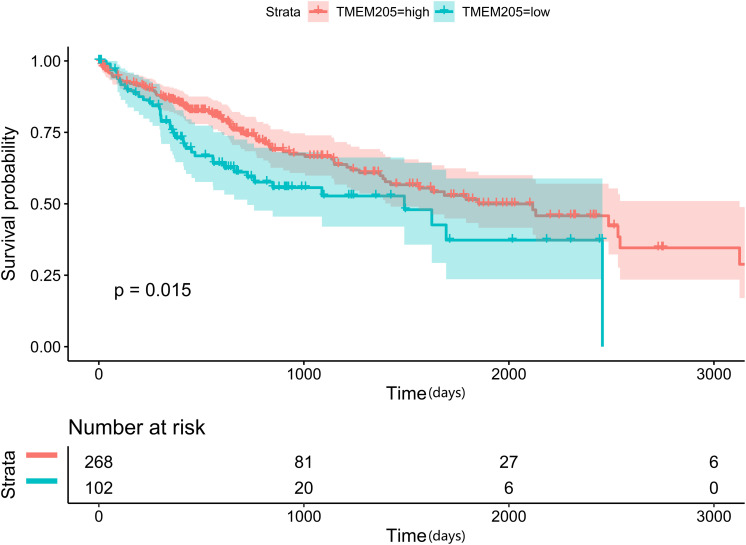
Low expression of TMEM205 is predictive for overall survival (OS) in hepatocellular carcinoma (HCC) patients from TCGA database.

**FIGURE 5:**
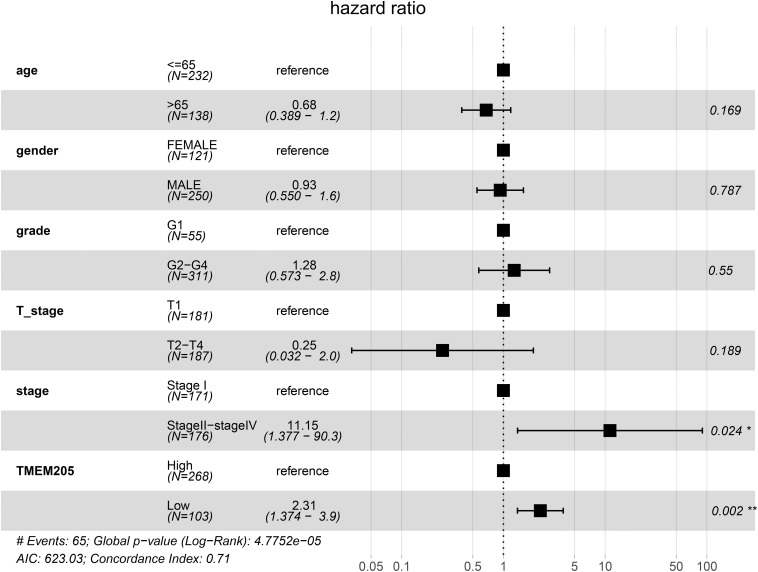
Low expression of TMEM205 is an independent prognostic factor for disease-specific survival (DSS) in HCC patients from TCGA database.

**FIGURE 6:**
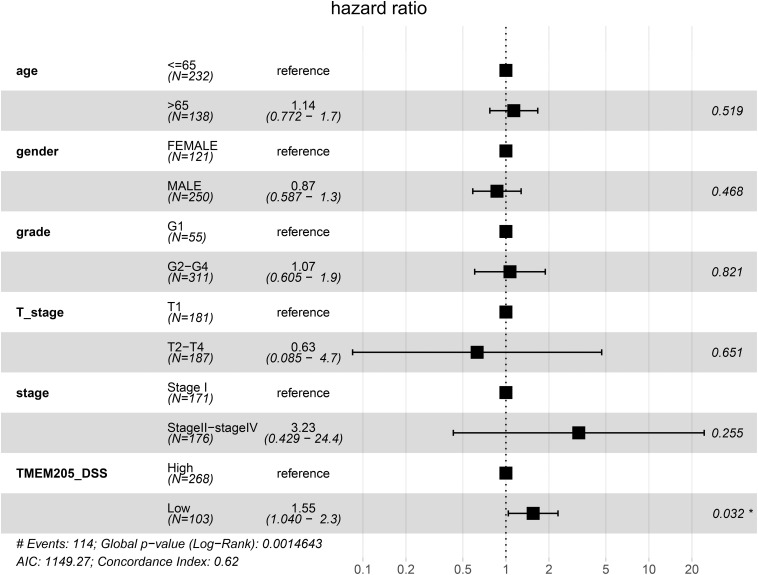
Low expression of TMEM205 is an independent prognostic factor for overall survival (OS) in HCC patients from TCGA database.

Through *t*-test with the TCGA RNA-seq datasets, we found differential expression of *TMEM205* in 50 paired tumor and normal tissues (*p* < 0.001; Figure 2). Using Kaplan–Meier analysis and univariate Cox regression analysis with the TCGA RNA-seq datasets, low *TMEM205* expression in tumor tissues was found to predict poor disease-specific survival (DSS; *p* = 0.005) in HCC patients (Figure 3). Low *TMEM205* expression was also associated with poor overall survival (OS; *p* = 0.015; Figure 4) and poor progression-free interval (PFI; *p* = 0.040) in HCC patients. To control for potential confounding clinical factors (age, gender, pathologic stage, pathologic T stage, and pathologic grade), we performed multivariate Cox regression analysis and confirmed that low *TMEM205* expression independently predicted poor DSS (*p* = 0.002; Figure 5) and poor OS (*p* = 0.032; Figure 6), but not poor PFI (*p* = 0.082).

### TMEM205 Expression Significantly Influences the Distribution of Immune Cell Subsets in the TME

**FIGURE 7:**
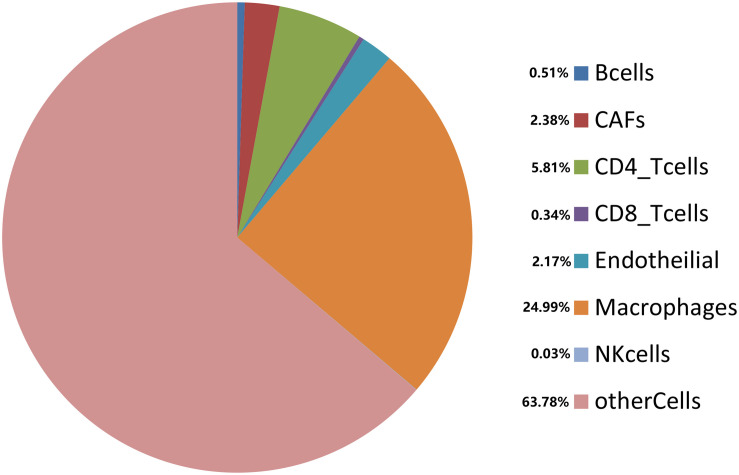
Estimated proportions of different types of immune cells in tumor tissues from TCGA database.

**FIGURE 8:**
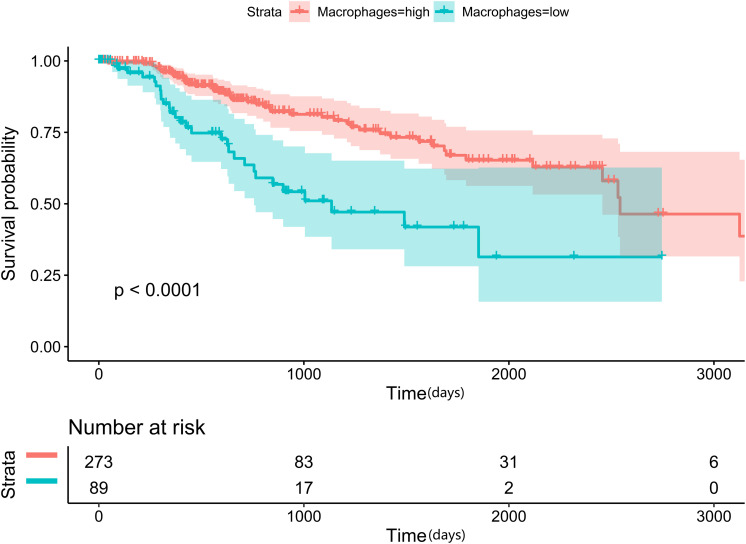
Low proportion of macrophages in tumor tissues is an independent prognostic factor for disease-specific survival (DSS) in HCC patients from TCGA database.

Cancer cells interact with various types of immune cells and stromal cells in the TME throughout all stages, from early carcinogenesis to tumor progression and metastasis (Hanahan and Weinberg, 2011). We used the efficient EPIC tool to estimate the proportions of cancer and immune cell types based on tumor mRNA expression data (Racle et al., 2017; Figure 7). Macrophages made up approximately 24.99%, while CD4+ and CD8+ T cells comprised 5.81 and 0.34%, respectively. Cancer-related fibroblasts accounted for 2.38%, endothelial cells accounted for 2.17%, and natural killer cells accounted for 0.03%. A total of 63.78% of the tumor tissues comprised other cells, which mostly consisted of cancer cells and other immune cells. Using the Kaplan–Meier survival curve and univariate Cox regression analysis, we found that a low proportion of macrophages was associated with poor DSS (*p* < 0.001) in HCC patients (Figure 8).

**FIGURE 9:**
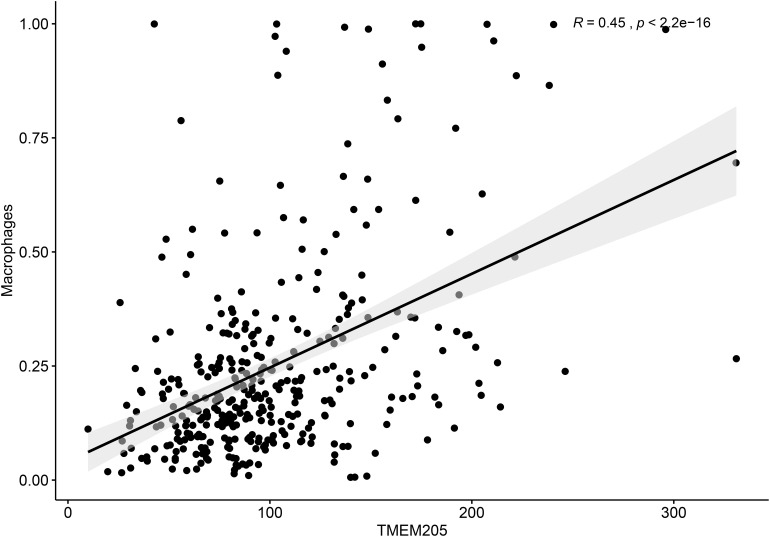
Expression of TMEM205 shows significant correlation with proportion of macrophages in tumor tissues from TCGA database.

Additionally, *TMEM205* expression in tumor tissues was significantly associated with the proportion of macrophages (Pearson *r* = 0.45, *p* < 0.0001; Figure 9). Combined with the above results, this indicates that the prognostic role of *TMEM205* expression might be explained by alterations in the distribution of immune cell subsets in the TME.

### TMEM205 Expression Is Associated With Decreased Numbers of Immunosuppressive Cells (M2 Macrophages and Tregs) and Infiltration of CD8+ T Cells Into the TME

**FIGURE 10:**
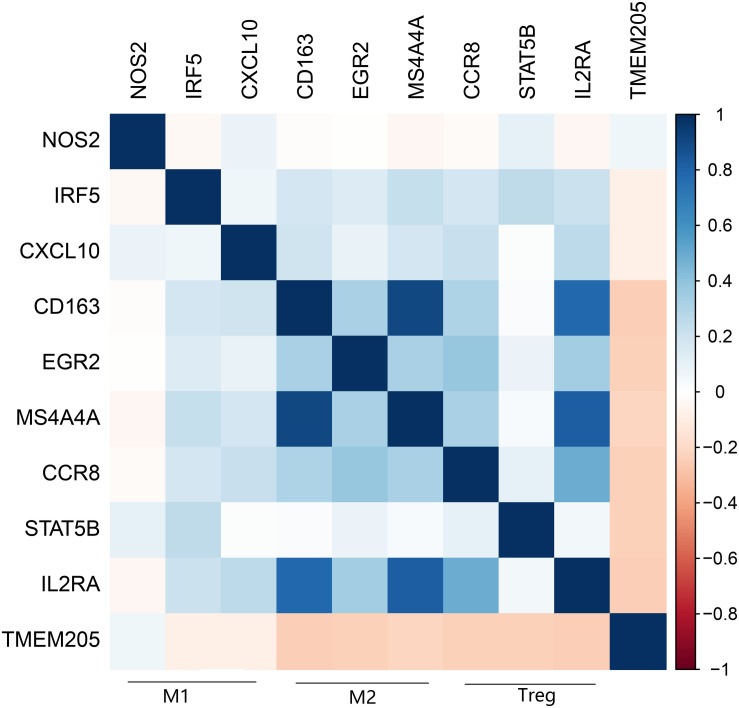
Expression of TMEM205 is significantly negatively correlated with markers of M2 macrophages (CD163, EGR2, and MS4A4A) and Treg (CCR8, STATA5B, and IL2RA) but is not correlated with markers of M1 macrophages (NOS2, IRF5, and CXCL10) from TCGA database. The color in the square reflects the correlation coefficient between square’s abscissa and ordinate.

**FIGURE 11:**
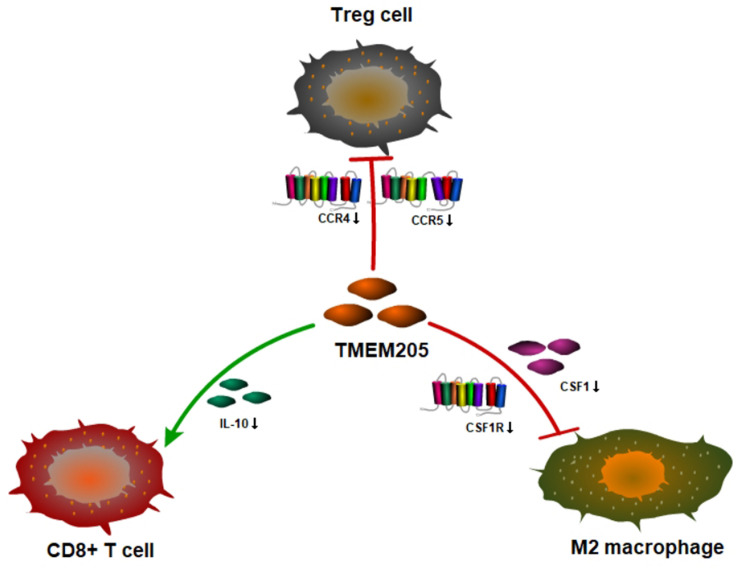
Hypothesis figure for the correlation between TMEM205 expression and immune cell distribution.

A recent study showed that the transformation of monocytes into specific CD163+ M2-like macrophages was induced by colony-stimulating factor 1 (CSF1) (Papin et al., 2019). Inhibition of CSF1 receptor (CSF1R) on TAMs did not kill the TAMs but caused them to repolarize to a tumoricidal state (Quail and Joyce, 2013). We found that *TMEM205* expression was negatively correlated with *CSF1* expression (Pearson *r* = −0.27, *p* < 0.0001) and *CSF1R* expression (Pearson *r* = −0.23, *p* < 0.0001). *TMEM205* expression was also negatively correlated with the expression of M2 macrophage markers (*CD163*, *EGR2*, and *MS4A4A*) (Jablonski et al., 2015; Sanyal et al., 2017; Terai et al., 2018; Figure 10). Additionally, *TMEM205* expression was negatively correlated not only with the expression of the chemokine receptors *CCR4* (Pearson r = −0.20, *p* < 0.0001) and *CCR5* (Pearson *r* = −0.25, *p* < 0.0001) (Kurose et al., 2015; Jiao et al., 2019), which are responsible for Treg migration to the TME, but also with the Treg markers (*CCR8*, *STAT5B*, and *IL2RA*) (Burchill et al., 2008; Nishikawa and Sakaguchi, 2014; Villarreal et al., 2018; Figure 10). Finally, we found that *TMEM205* expression appeared to inhibit *IL-10* expression (Pearson *r* = −0.22, *p* < 0.0001), while *TMEM205* expression was positively correlated with the proportion of CD8+ T cells in tumor tissues (Pearson *r* = 0.26, *p* < 0.0001). Together, these data suggest that *TMEM205* expression might inhibit M2 macrophage polarization, inhibit Treg recruitment, and facilitate CD8+ T cell infiltration into tumor tissues, thereby improving the patients’ prognosis, which is summarized in Figure 11.

### Validation of TMEM205 in the ICGC Cohort

**FIGURE 12:**
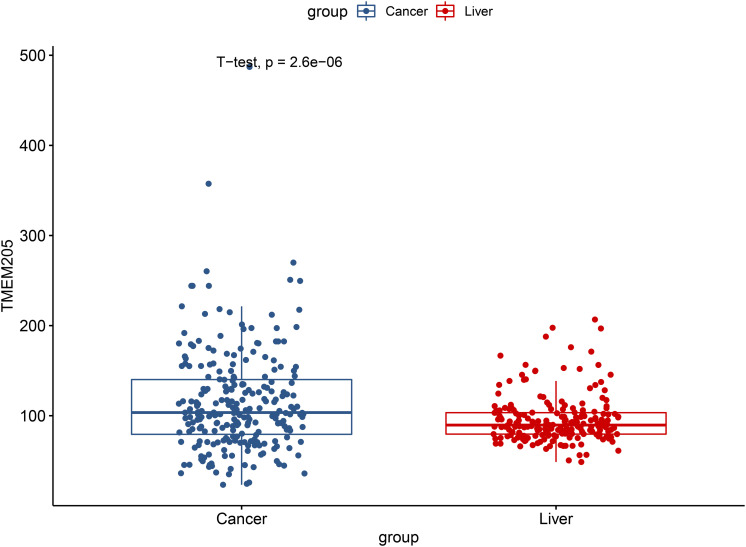
Differential expression of TMEM205 between normal and tumor tissues in ICGC database.

**FIGURE 13:**
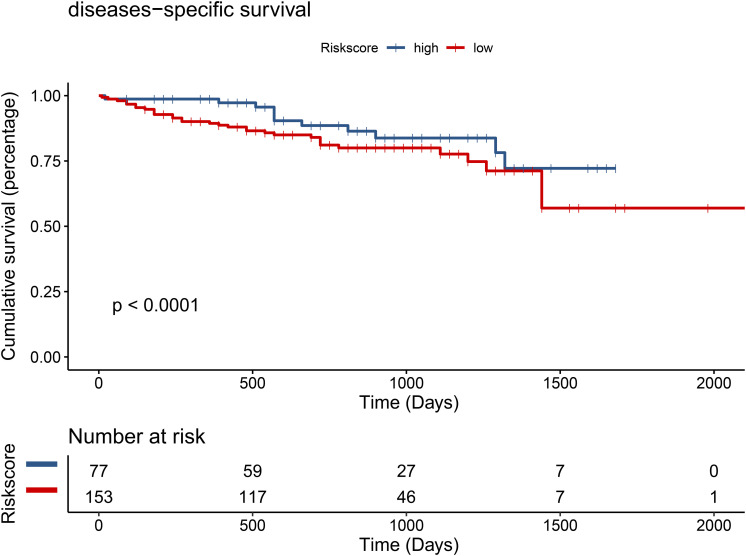
Low expression of TMEM205 is predictive for survival in hepatocellular carcinoma (HCC) patients from ICGC database.

We validated the significant difference of *TMEM205* expression between normal tissues and tumor tissues in the ICGC cohort (*p* < 0.001; Figure 12). Low expression of *TMEM205* was also found to be associated with poor survival (*p* < 0.001; Figure 13).

## Discussion

Previous studies have reported associations between many members of the TMEM family and tumor progression (Qiao et al., 2016; Gao et al., 2017; Wang et al., 2017; Akkafa et al., 2018; Song et al., 2018), but the role of *TMEM205* was not previously established. In the current study, we not only found significant difference in *TMEM205* expression between normal tissue and tumor tissue (*p* < 0.001) but also demonstrated that low *TMEM205* expression was independently associated with poor OS (*p* = 0.032) and DSS (*p* = 0.002) in HCC patients from the TCGA cohort. A significant differential expression of *TMEM205* was also observed (*p* < 0.001) and low *TMEM205* expression still predicted poor survival (*p* < 0.001) in HCC patients from the ICGC cohort, which validated that *TMEM205* expression was an independent prognostic factor in HCC.

Next, we found that having a low proportion of macrophages in the TME predicted poor DSS (*p* < 0.001) in HCC patients. This result is similar to the finding by Li et al. that a high proportion of intratumoral CD68+ TAMs in HCC patients predicted improved survival (Li et al., 2009). We also found a positive correlation between *TMEM205* expression and the proportion of macrophages in tumor tissues (Pearson *r* = 0.45, *p* < 0.0001). However, the role of TAMs in tumor evolution seems to be complex. Classical M1 macrophages and alternatively activated M2 macrophages are two major functional phenotypes of TAMs, and they promote tumoricidal responses and increase tumor progression, respectively (Gordon and Taylor, 2005; Wang et al., 2010). Confirming this, inhibiting M2 macrophage polarization was recently shown to lead to impaired tumor growth and impaired immune evasion (Ye et al., 2018). In the current study, we could not determine the proportions of more precise cell subtypes such as M2 macrophages, due to the restricted functions of the EPIC tool, but we identified negative correlations with M2 macrophage markers (*CD163*, *EGR2*, and *MS4A4A*). *CD163* is a type of hemoglobin scavenger receptor that indirectly contributes to the anti-inflammatory response (Etzerodt and Moestrup, 2013), and it is well known as a specific M2 marker, based on *in vitro* and *in vivo* evidence (Ino et al., 2013; Erlandsson et al., 2019). *EGR2* expression on macrophages increases after stimulation with M2-like stimuli (*IL-4* or *IL-13*) (Veremeyko et al., 2018). *MS4A4A* may serve as a novel cell-surface marker for M2 macrophages (Sanyal et al., 2017). We also observed that *TMEM205* expression was negatively correlated with *CSF1* expression (Pearson *r* = −0.27, *p* < 0.0001) and *CSF1R* expression (Pearson *r* = −0.23, *p* < 0.0001). A previous study showed that the downstream signaling pathway of *CSF1R* was responsible for the polarization of TAMs to an immunosuppressive M2 phenotype (Ramesh et al., 2019). Thus, there might also be a negative correlation between *TMEM205* expression and the proportion of M2 macrophages in tumor tissues.

Additionally, we found a negative correlation between *TMEM205* expression and Treg markers (*CCR8*, *STAT5B*, and *IL2RA*). *CCR8* is a chemokine receptor mainly expressed on Tregs and it plays a critical role in Treg-mediated immunosuppression (Villarreal et al., 2018). *STAT5B* deficiency is associated with decreased *FOXP3* expression and Treg levels (Kanai et al., 2012). *IL2RA* (also known as *CD25*) is also a marker that is used to quantify Treg levels in individuals (Yu et al., 2012). Furthermore, we found a negative correlation between *TMEM205* expression and the chemokine receptors *CCR4* (Pearson *r* = −0.20, *p* < 0.0001) and *CCR5* (Pearson *r* = −0.25, *p* < 0.0001), which are responsible for Treg migration to the TME (Kurose et al., 2015; Jiao et al., 2019). These results suggested a negative correlation between *TMEM205* expression and the proportion of Tregs in tumor tissues. It is well known that Tregs inhibit antitumor immunity and are involved in tumor development (Ohue and Nishikawa, 2019).

We also found a negative correlation between *IL-10* expression and *TMEM205* expression (Pearson *r* = −0.22, *p* < 0.0001). High IL-10 levels predict poor breast cancer prognosis and the IL-10 secreted by TAMs can inhibit T cell effector functions and induce regulatory functions that recruit Tregs (Noy and Pollard, 2014; Ahmad et al., 2018). Therefore, it was not difficult to find a positive correlation between *TMEM205* expression and the proportion of CD8+ T cells (Pearson *r* = 0.26, *p* < 0.0001).

In the last few decades, great progress has been made in the understanding of HCC biology and immunotherapy, but the therapeutic effects of anti-cytotoxic T-lymphocyte-associated protein 4 (CTLA4) and anti-PD-1 immunotherapy remain unsatisfactory (Sangro et al., 2013; El-Khoueiry et al., 2017; Zhu et al., 2018). Additionally, a significant percentage of patients eventually develop resistance to immunotherapy and many patients are only partial responders (Topalian et al., 2012; Hamid et al., 2013; Zou et al., 2016). Among all the possible reasons, the disturbance of local immunosuppressive cells in the TME might be one of the most important ones. Kato et al. showed that a decreased M2 population and an increased M1/M2 ratio tended to be associated with a better therapeutic effect regarding anti-PD-1 therapy (Kato et al., 2019). High Tregs levels also impaired the response to anti-PD-1/PD-L1 therapy (causing a poor immunologic response against the tumor) (Bettelli et al., 2006), and strategies involving targeting Tregs lead to improved responsiveness to anti-PD-1/PD-L1 therapy (Arce Vargas et al., 2017; Taylor et al., 2017). Nowadays, combination therapy is the major tumor therapy approach, and a recent study found that an anti PD-1 drug (atezolizumab) combined with an anti-vascular endothelial growth factor drug (bevacizumab) led to encouraging antitumor activity and safety in patients with unresectable HCC (Finn et al., 2020). In this study, we found that *TMEM205* expression was not only associated with HCC prognosis but also with decreased levels of immunosuppressive cells (M2 macrophages and Tregs) and infiltration of CD8+ T cells into the TME. This suggests that *TMEM205* may be useful as part of combination therapy regimens.

Several limitations in our study should be recognized. First, due to the restricted functions of the EPIC tool, we could not determine the proportions of more specific subtypes of immune cells (such as M2 macrophages or Tregs). Instead, we could only assess associations with corresponding cell markers, which might have produced bias. Also, the clinical data on the immunotherapy administered to HCC patients were unclear, which prevented a more detailed analysis from being conducted.

In summary, we first confirmed that *TMEM205* expression is an independent prognostic factor in HCC patients, and we then found that the antitumor effect might be explained by reducing the numbers of immunosuppressive cells (M2 macrophages and Tregs) and facilitation of CD8+ T cell infiltration into the TME. These results suggest that TMEM205 may be useful as a therapeutic agent.

## Data Availability Statement

The RNA sequencing data for this study can be found in the TCGA data portal (https://portal.gdc.cancer.gov/). The clinical data can be found in datasets of UCSC Xena (https://xenabrowser.net/datapages/).

## Author Contributions

YM conceptualized the study. JR, XW, and XZ were responsible for investigation and statistical analysis. JR and XW wrote the original manuscript. JR and RD reviewed and edited the manuscript. YM was responsible for funding acquisition. All authors contributed to the article and approved the submitted version.

## Conflict of Interest

The authors declare that the research was conducted in the absence of any commercial or financial relationships that could be construed as a potential conflict of interest.

## Footnotes

1 https://gfellerlab.shinyapps.io/EPIC_1-1/
